# Effect of local versus global risk and normative information on voluntary quarantine adoption: protocol for a 2×2 randomised controlled trial leveraging app-based epigames across diverse congregate settings (The Epi-Q Study)

**DOI:** 10.1136/bmjopen-2026-116726

**Published:** 2026-10-08

**Authors:** Estelle McCool, Winnie W. Mkandawire, Salihu S. Musa, Shi Chen, Laura Moley, Gwen Robbins Schug, Nick Bowman, Sarah A. Nowak, John M. Drake, John Drury, Adam Fowler, Melinda C. Mills, Christophe Fraser, Luca Ferretti, Ben Swallow, Jasmina Panovska Griffiths, Andrés Colubri

**Affiliations:** 1 Pandemic Sciences Institute, University of Oxford, Oxford, UK; 2 Health Data Research UK, London, UK; 3 University of Oxford Brasenose College, Oxford, UK; 4 Department of Genomics and Computational Biology, UMass Chan Medical School, Worcester, Massachusetts, USA; 5 Broad Institute of Harvard and MIT, Cambridge, Massachusetts, USA; 6 School of Data Science, University of North Carolina, Charlotte, North Carolina, USA; 7 Department of Geography and Geospatial Sciences, Kansas State University, Manhattan, Kansas, USA; 8 Department of Biology, University of North Carolina Greensboro, Greensboro, North Carolina, USA; 9 Newhouse School of Public Communications, Syracuse University, Syracuse, New York, USA; 10 Larner College of Medicine, University of Vermont, Burlington, Vermont, USA; 11 University of Georgia Odum School of Ecology, Athens, Georgia, USA; 12 School of Psychology, University of Sussex, Brighton, UK; 13 St Peter’s College, University of Oxford, Oxford, UK; 14 Nuffield Department of Population Health, University of Oxford, Oxford, UK; 15 School of Mathematics and Statistics, University of St Andrews, St Andrews, UK; 16 The Queen’s College Oxford, Oxford, UK

**Keywords:** Research Design, Mobile Applications, Epidemics, INFECTIOUS DISEASES, Social Interaction

## Abstract

**Abstract:**

**Introduction:**

We describe the protocol for the EpiGame-Quarantine (Epi-Q) Study, which will apply an experimental epidemic game (‘epigame’) to investigate quarantine-related attitudes and behaviours. Epigames are field experiments in which participants make behavioural decisions during a simulated epidemic in naturalistic settings. In this study, the epigame is operationalised via a smartphone app that simulates transmission of a ‘digital pathogen’ over Bluetooth, allowing for real-time data capture. This approach offers a novel tool for observing decision-making within social networks under dynamic risk and normative pressure. Consequently, it enables the collection of empirical data on the social, psychological and practical determinants of compliance with non-pharmaceutical interventions such as quarantine. Unlike existing methods that rely on static cross-sectional surveys or observational data, Epi-Q captures dynamic decision-making within real-life networks. This is crucial for studying behaviours like quarantine, in which compliance is not merely an individual calculation but is shaped by pre-existing and evolving social structures, including peer pressure, trust in institutions and exposure to misinformation. Epi-Q employs a quantitative mixed-method design to untangle the complex interplay between social norms and risk perception, along with their interactions on behavioural responses.

**Methods and analysis:**

We will recruit at least 150 participants via a custom-made smartphone app capable of measuring proximity between them using Bluetooth. We will randomly allocate these participants (via network-aware clustering) into a 2×2 experimental design: (i) local versus global risk information and (ii) local versus global norms. ‘Local’ information is defined as data derived from participants’ first-degree contacts in the Bluetooth proximity network, whereas ‘global’ information refers to aggregate data from the entire experimental group. Participants will play the epigame over a period of several weeks (1–3), making daily quarantine decisions within the app that incur costs (loss of in-game points for infection and loss of opportunities while quarantining) and benefits (points from remaining active), while Bluetooth proximity sensing samples the contact network of participants and drives simulated transmission of a digital pathogen among the phones. Data collection will include in-app behavioural logs, pre-game, mid-game and post-game surveys (attitudes and risk perception) and optional qualitative debrief interviews with a subset of participants. Primary outcomes will be frequency and timing of quarantine adoption; secondary outcomes will include attitudinal shifts, perceived risk and the consistency of real-world versus in-game intentions. We will use a range of methods including mixed-methods synthesis, difference-in-differences modelling, social network analysis and correlation of survey and behavioural measures to analyse the data and assess external validity.

**Ethics and dissemination:**

The study protocol has been reviewed and approved by UMass Chan IRB, with ID 2465. Results will be disseminated in peer-reviewed journals, conferences and through the project and collaborators’ websites.

**Study registration:**

The project plan of the study can be found at https://osf.io/7ds6w

**STRENGTHS AND LIMITATIONS OF THIS STUDY**

- We introduce the EpiGame-Quarantine (Epi-Q) study as a novel virtual randomised controlled trial via an experimental game (‘epigame’) run with the Epigames app.
- The Epi-Q study allows for causal inference of behavioural drivers in a dynamic, networked environment.
- Mixed-methods approach (attitudinal surveys, behavioural game data and network data) will triangulate findings.
- External validity is explicitly tested by mapping real-world and in-game beliefs.

## Introduction

### Background and rationale

 Voluntary quarantine and isolation are among the most effective measures for reducing the spread of infectious disease,^1 2^ but adoption is often incomplete and variable. Behavioural science research highlights the role of perceived risk, social norms and self-efficacy in shaping quarantine decisions.^3–5^ From an anthropological perspective, these individual-level factors are embedded within a broader sociocultural context.^6 7^ Structural inequalities and identity-based stigma can create significant barriers to compliance as public health measures are often experienced differently across social groups.^8 9^ Game-theoretic models predict complex feedback loops between prevalence and protective behaviour, generating epidemic waves that resemble real-world patterns.^10–12^ Despite these conceptual advances, empirical probing of such behavioural factors and dynamics, particularly the social forces that inhibit or promote compliance, remains limited.

This study is designed to experimentally test predictions from construal level theory^13^ (regarding local vs global information) and social contagion theory^14^ (regarding the diffusion of behavioural norms) while accounting for anthropological insights on how factors such as social identity can modulate these influences. Experimental epidemic games (hereafter ‘epigames’) are field experiments^15^ where participants make real-time decisions in response to a simulated epidemic in naturalistic congregate settings. They offer a novel approach for controlled experimentation on dynamic behavioural responses. Unlike surveys, games embed participants in evolving, incentive-driven scenarios that can simulate the social pressures and trade-offs inherent in quarantine decisions. Furthermore, games allow manipulation of key informational variables to explore effects of possible interventions of behavioural changes, a crucial distinction from observational studies. For example, games can be used to calculate the transmission risk and potential inequity in infectious diseases outbreak^16^ or quantification of the impact of physical distancing in controlling outbreaks.^17^

### Objectives

This protocol describes the design of the Epi-Q study, an epigame aimed at the following:

1. Quantifying the effects of dynamic risk information and social norms on quarantine behaviour.
2. Testing whether local versus global information differentially influences compliance.
3. Testing the degree to which in-group-bonds can amplify social influence
4. Measuring the alignment (or ‘attitude–behaviour gap’) between self-reported intentions and revealed in-game behaviour.
5. Developing methods to assess external validity of epigames as a customizable research framework.

## Methods and analysis

### Study design

The Epi-Q study is a four-stage virtual randomised control trial (RCT) epigame conducted via a smartphone app custom-built for this purpose, allowing for the simultaneous and anonymised collection of contact, behavioural and attitudinal data from participants. We will be using the *Epigames* app,^18^ which builds on the existing Operation Outbreak (OO) app.^19^ OO uses Bluetooth proximity sensing to enable experiential learning simulations on infectious disease.^20^ The *Epigames* app uses the same proximity sensing technology as OO, based on the open-source Herald project,^21^ to sample high-resolution social contact networks between participants. In addition, the Epigames app prototypes a customizable tool for gamified behavioural research. It offers a survey module to create multilingual pre-game, mid-game and post-game attitudinal questionnaires for participants, visualisation features to present contextual data to participants within the app itself, a library of interventions such as contact tracing and quarantine to construct specific gamified intervention scenarios, a pathogen simulation engine for realistic transmission dynamics during the study and randomised assignment of participants to control and intervention groups. This infrastructure can simulate detailed, real-time person-to-person pathogen spread and epidemic dynamics via Bluetooth-based proximity sensing and tracking participants’ health behavioural responses and attitudinal preferences. We are planning to run the Epi-Q study as a series of 2–3 epigames across settings and cohorts over the period from March 2027 to December 2027.

During the epigame in the Epi-Q study, participants may choose to ‘quarantine’ each day by selecting a button in the app. Although in-game quarantine does not correspond to physical isolation, it is designed to provide ‘action fidelity’ by introducing tangible consequences for behavioural choices. First, in-app quarantine temporarily suspends the Bluetooth contacts of the participant, protecting them from infection as real-life quarantine would do. Second, the app imposes an explicit cost–benefit structure where the safety of quarantine is weighed against the loss of in-game points (analogous to lost social/economic opportunities), which can be tied to real-world financial incentives. This allows the study to capture responses to different informational interventions in relation to three key determinants of quarantine compliance: (i) personal attitudes, (ii) social norms and (iii) perceived risk.^5^ Self-efficacy will also be measured, but given that quarantine is much easier to perform in the game than in real life, it will not be a primary focus of analysis.

Participants accumulate points for each day they spend outside quarantine, reflecting the individual-level benefits of continuing work, socialising and other normal activities. They lose points if they become infected by the digital pathogen. The cost of infection (points lost) is individually randomised, and participants are informed of this cost at the start of the game.

To ensure fair incentives, points are converted into lottery tickets for entry into a prize draw (eg, gift cards), with the number of tickets proportional to the participant’s final score. This design prevents ‘winner-takes-all’ dynamics that could otherwise distort health decision-making and behavioural responses during the game.

### Setting and participants

Participants will be recruited from diverse congregate settings, including college campuses, professional conferences, office workspaces and via national registers for volunteer recruitment such as ResearchMatch.^22^ Recruitment will take place remotely via digital methods (email invitations, study website, QR codes and online postings) and physical flyers. We plan to conduct at least three instances of the Epi-Q study, one at a college campus, another at a professional conference and a third through ResearchMatch. This would generate data from distinct populations: young adults (college), working professionals (conference) and general members of the population (ResearchMatch). Irrespective of the setting and recruitment method, each instance of the study will be carried out as an epidemic simulation in a real-life environment and sampling physical contacts between participants.

Eligible participants must be adults (≥18 years) who own a smartphone and have access to the internet. Non-English speakers may participate if a translated version of the app is available. Exclusion criteria include individuals under 18, those unable to provide informed consent or those without access to the required technology. Vulnerable populations such as prisoners and cognitively impaired adults will be excluded. Pregnant women may participate, and there is no real-world health risk involved. Students may be included, and no study team member has influence over their academic standing.

Participation is voluntary and expected to last 1–3 weeks per game, with a target enrolment of 200–500 participants per game (minimum~150 required for analysis). We have plans for compensation to participants, but individual compensation will vary depending on the specific game design. In some studies, participants will receive digital gift cards (typically US$5–20) delivered through the app; in others, a lottery system will be used or no compensation provided if financial incentives could bias behaviour.

Informed consent is obtained electronically within the app. Participants are presented with an onboarding process describing the study, data use and privacy protections, followed by an informed consent screen requiring acknowledgement via checkbox and timestamp. No personally identifiable information (eg, name, email, location and phone number or IP address) is collected. Each participant is instead assigned a random 128-bit universally unique identifier, which anonymises all app data. Participants may withdraw at any time via a clearly labelled option in the app, which also permanently deletes their data.

### Patient and public involvement

None.

### Intervention and study stages

The experiment will occur in four discrete stages (figure 1).

**Figure 1:**
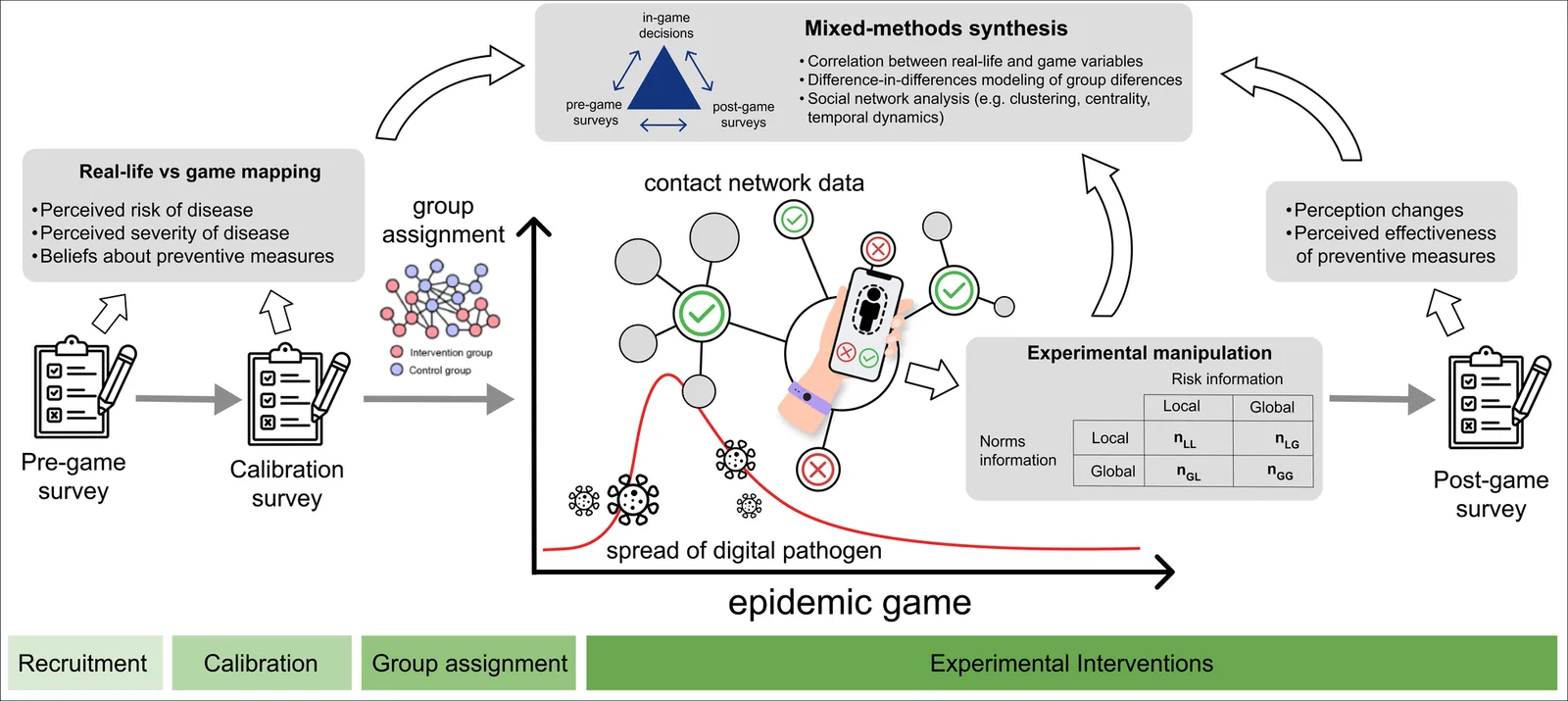
*Diagram summarising all stages of the Epi-Q study and the procedures to be performed in each*. Epi-Q*,* EpiGame-Quarantine.

#### Stage 1: recruitment (30 days)

Participants will be invited to download the app and enter a unique code provided by the research team. After providing consent digitally, they will complete a baseline survey (online supplementary S1) assessing real-world perceptions of risk, severity, quarantine efficacy, self-efficacy and social norms, as well as a measure of social identification with peers. Following this, participants will receive onboarding instructions and visual materials about the study. The app will remain inactive during this recruitment window, allowing participants to enrol over a 1-month period.

#### Stage 2: calibration (2 days)

Participants will receive a reminder notification when the game is ready to begin; afterwards, they will complete a second survey (online supplementary S2) framed in terms of the upcoming game scenario, allowing for mapping of real-world versus in-game attitudes. During this stage, the app will remain ‘paused’, with no infections occurring, but will continuously sample Bluetooth proximity data to calibrate distance estimation and infer the baseline contact network among participants. These data are used both to validate app performance and, when participants are recruited from the same population, to inform subsequent randomisation.

#### Stage 3: group assignment (3 days)

Using contact data from stage 2, participants will be assigned to one of four experimental groups via a graph cluster randomisation algorithm designed to minimise spillover across groups.^23^ If groups are recruited from distinct populations (eg, separate campuses), this step is not required. After randomisation, initial infections are seeded randomly across groups, and disease spread is observed for 3 days without additional interventions. This baseline period ensures comparability of epidemic dynamics and quarantine adoption across groups before interventions begin.

#### Stage 4: intervention (between 7 and 21 days)

This stage will last for a pre-determined period of 14 days to standardise exposure across participants and capture weekly periodicity in contact networks, though this may be adjusted to 7 or 21 days in specific deployments to test the effect of epidemic duration on behaviour, or until a preset prevalence is reached. During the main study period, participants receive group-specific informational cues in a 2×2 factorial design: (i) local versus global risk information (infection incidence among close contacts vs population-level prevalence) and (ii) local versus global norm information (quarantine uptake among close contacts vs overall population). Each day, participants decide whether to quarantine, with payoffs structured as points gained for staying active, points lost if infected and points foregone while quarantining. To empirically calibrate these values, a pre-study ‘willingness to accept’ pilot survey^24^ will identify the median acceptance threshold, defined as the specific reward gap at which 50% of participants switch from the safe ‘quarantine’ option to the risky ‘active’ option. This threshold will be used to parameterise the payoff structure, ensuring the decision dilemmas are set at a point of maximum behavioural tension.

At the conclusion of the game, participants complete a final survey (online supplementary S3) assessing post-game perceptions, perceived cognitive load and reflections on consistency between in-game and real-life behaviour.^25–28^

## Outcomes

### Primary outcome

1. Total days quarantined per participant during the intervention stage (table 1). This outcome directly captures the behavioural response to the experimental manipulations of social norm information and risk information in the 2×2 factorial design (local vs global risk×local vs global norms).

**Table 1:** Factorial 2×2 design with the output measured in each case: N_nl,rl_, N_nl,rl_, N_ng,rl_ and N_ng,rg_ represent the number of participants who adopt quarantine within each intervention group

		Risk information (# of infections)	
		Local	Global
Norms information (# of quarantine adopters	Local	N_nl,rl_	N_nl,rl_
	Global	N_ng,rl_	N_ng,rg_

### Secondary outcomes

1. **Infection incidence in the simulated epidemic.** Daily simulated epidemiological states (susceptible, exposed, asymptomatic infectious, symptomatic infectious, recovered or deceased) will be logged for each participant, allowing comparison of infection outcomes across intervention groups.
2. **Survey-based attitudinal changes.** Changes in risk perception, perceived severity, self-efficacy and perceived social norms between pre-game (S1), calibration/game-framed (S2) and post-game (S3) surveys.
3. **Consistency of real-world versus in-game attitudes.** Correlation between baseline real-world attitudes (S1) and corresponding in-game attitudes (S2), as well as correlation between survey responses and observed in-game choices (‘attitude–behaviour gap’).
4. **Perceived difficulty and cognitive load.** Participant ratings (S3) of how easy or difficult it was to process information provided during the game.
5. **Peer influence thresholds.** Derived from the fraction of infected or quarantining peers required to trigger quarantine adoption by an individual, as estimated from high-resolution contact and decision data.
6. **Effect of informational proximity.** Differences in quarantine uptake under local versus global information conditions (first-degree contacts vs aggregate population), testing the hypothesis that socially proximal information exerts stronger influence on behaviour (as suggested by construal-level theory and egocentric bias in risk perception).
7. **Network-derived variables.** A time-varying graph to represent the social contact network over the course of the epigame can be constructed from Bluetooth proximity data. This will allow us to calculate measures such as clustering, centrality and reachability to examine how network position modulates responses to risk and norm information.

### Exploratory outcomes

1. Effect of individualised payoff structures (randomly assigned infection cost) on participants’ willingness to quarantine.
2. Post-game reflections on behavioural realism (participants’ self-reported similarity of in-game vs real-life health decision-making on quarantine).

### Predictions (hypothesis)

**H1 (risk information):** the scope of risk information (local vs global) will significantly affect quarantine compliance. Specifically, we hypothesise that socially proximal (local) risk information will exert a stronger influence on compliance than aggregate (global) population-level statistics.

**H2 (normative information):** the scope of normative information (local vs global) will significantly affect quarantine compliance. We hypothesise that observing quarantine behaviours among close contacts (local norms) will drive higher compliance than observing global population statistics.

**H3 (attitude–behaviour gap):** there will be a measurable discrepancy between self-reported intentions (surveys S1/S2) and revealed in-game quarantine behaviour, which may vary by experimental condition.

**H4 (peer influence thresholds)**: individuals with higher stated willingness to quarantine in baseline surveys will exhibit lower behavioural thresholds for adoption (requiring fewer infected/quarantining peers to trigger action).

### Data collection and management

The app records (i) survey responses, (ii) daily quarantine decisions, (iii) Bluetooth proximity data and (iv) simulated epidemiological states for each participant. Data do not contain any personal identifiers and are stored in a secure cloud database.

### Sample size

Power analyses were conducted to estimate the number of participants required for the 2×2 factorial design. Detecting a large effect size (OR ≈ 2, Cohen’s *d* ≈ 0.38) would require approximately 150 participants, while detecting more moderate effects (*d* ≈ 0.15) would require >500 participants. We therefore plan to recruit at least 150 participants, with larger samples pursued where feasible depending on available populations.

The basis for this analysis draws on evidence from diffusion of innovation theory and social network studies of peer influence. Research on adolescent smoking and drinking suggests that peer behaviour is associated with adjusted ORs (AORs) of approximately 2.0–3.0 when measured as proportions, and 1.2–1.4 when measured as counts, while network intervention studies have also reported effect sizes corresponding to ~14 percentage point increases in outcomes, considered large by most standards.^29–33^ These effects are relatively stable across a range of peer-influenced behaviours and can reasonably be extended to the present context of quarantine adoption.

To translate ORs into standardised effect sizes, we applied the formula proposed by Chinn.^34^ Using this formula, OR ≈ two yields *d* ≈ 0.38 (a large effect), while OR ≈ 1.4 yields a more moderate effect size. Assuming an effect size of similar magnitude for quarantine adoption, a sample size of ~150 would be sufficient for adequate statistical power. However, to detect smaller effects (~0.1), a substantially larger number of participants (>1500) would be required. This is feasible, as pilot studies with the OO and *Epigames* apps at conferences and on college campuses involved at least 100 participants and nearly 1000 in some instances.^35^

### Statistical analysis

We will employ a combination of descriptive, inferential and network-based approaches to analyse data from the Epi-Q study. Analyses will focus on measuring the correlation between in-game behaviour and real-world attitudes, quantifying peer influence and estimating group-level intervention effects.

#### Mapping index between real-world and in-game attitudes

Responses from the pre-game survey (S1) and the calibration/game-framed survey (S2) will be analysed to compute a correlation-informed mapping index between real-life and in-game perceptions (eg, perceived infection risk, severity and efficacy of quarantine). This index will quantify the degree of indicator (behaviour) parallelism between real-world and simulated contexts and serve as a measure of external validity.^36^

#### Attitude–behaviour gap

To assess the discrepancy between stated intentions and revealed preferences, we will compare participants’ survey responses about their likelihood of quarantining with their actual in-game decisions (eg, Likert scale from S1 could be mapped to fraction of days quarantined, per participant). Analyses will explore whether the magnitude of this gap varies by game-specific factors, such as perceived infection risk or group assignment.

#### Peer influence modelling

In-game behavioural data will be analysed as revealed preferences in the presence of peer behaviour cues. We will test whether individuals with a high stated willingness to quarantine (S1/S2) exhibit lower behavioural thresholds for adoption (ie, requiring fewer infected or quarantining peers before choosing to quarantine). Logistic regression models will be constructed by including global information exposure and local network measures (eg, proportion of first-degree contacts quarantined) as predictors and daily binary decision to quarantine as outcome.

#### Group differences under intervention conditions

The factorial design allows causal inference regarding the effects of information type (local vs global; risk vs norms). We will estimate these effects using difference-in-differences (DID) models, comparing changes in quarantine uptake before and after intervention across groups. DID models will be specified as follows:

$\begin{array}{l} Y=\beta_{0}+\beta_{1}\times \text{time}+\beta_{2}\times \text{intervention}+\beta_{3}\\ \;\;\;\;\;\;\;\;\times \text{time}\times \text{intervention}+\beta_{4}\times \text{covariates}+\varepsilon \end{array}$

where *Y* is the number of quarantine days per participant. DID assumptions (parallel trends, stable group composition and limited spillover) will be checked explicitly. Spillover bias will be minimised by graph-cluster randomisation.

#### Exploratory analyses

- Network effects: we will incorporate network-derived metrics relating to the fundamental structure of the interaction network (eg, clustering coefficient and degree centrality distribution) and to the temporal structure of the network to test whether the underlying structure of social interactions influence responsiveness to interventions.
- Heterogeneity of treatment effects: subgroup analyses (eg, by baseline attitudes and demographic variables) will explore moderators of intervention effects.
- Convergence of mixed-methods data: in-game choices will be triangulated with survey responses and, where available, qualitative feedback to identify convergences or divergences in behavioural drivers. We will use established methods such as Braun and Clarke’s thematic analysis to seek out patterns of response.^37^

Quantitative analyses (data parsing, statistical modelling and testing) will be conducted in *R* or *Python*, with appropriate multiple-comparison adjustments (eg, Holm or Bonferroni) applied, while *NVivo* will be used for qualitative analyses. Code will be open-source and made publicly available.

## Discussion

### Relevance

The COVID-19 pandemic highlighted the importance of voluntary protective behaviours such as quarantine and self-isolation while revealing substantial variability in compliance. Social science insights from the pandemic revealed that stigma, conspiracy thinking and social isolation often acted as powerful countervailing forces against public health directives.^6 38 39^ Behavioural game theory has long predicted feedback loops between risk perception, social norms and epidemic dynamics, but empirical tests of these processes in naturalistic settings remain limited. The Epi-Q study addresses this gap by introducing epigames as a new methodology for capturing real-time behavioural decision-making within dynamic, networked contexts. Unlike conventional surveys, which capture hypothetical intentions, and observational studies, which lack experimental control, epigames allow manipulation of key informational variables in real time. The high-resolution behavioural and network data generated can inform epidemic models that better account for endogenous human behaviour and the social forces that constrain it, ultimately contribute to more realistic predictions of outbreak trajectories.

### Strengths and limitations

A major strength of the Epi-Q study is its integration of three key complementary data streams: (i) self-report surveys of attitudes and perceptions, (ii) in-game behavioural data collected under controlled experimental conditions and (iii) high-resolution proximity-based contact network data. This mixed-methods approach increases construct validity and allows triangulation across data sources. The study also directly addresses external validity by comparing real-world and in-game attitudes. The factorial design provides causal leverage to disentangle the influence of risk information and social norms, while the use of graph cluster randomisation reduces bias from spillover across groups.

However, the epigame study framework has limitations. First, although designed to simulate real-world health decision contexts, in-game choices may not fully generalise to actual quarantine behaviour in real epidemics (eg, people may play the game in ways that motivate them to be more adherent to rules than they would in real life). Second, attrition and variability in engagement may reduce effective sample size; we will address this through ongoing engagement monitoring and by applying an intention-to-treat principle, using sensitivity analyses (eg, multiple imputation or inverse probability weighting) to assess robustness to missing data. Third, the payoff structure, while designed to reflect real-world trade-offs, simplifies the complex costs and benefits of quarantine. Fourth, the reliance on smartphone Bluetooth sensing introduces technical noise in contact detection, though calibration procedures aim to mitigate this. Finally, while we will analyse the diffusion of behaviour through the network, the study is designed to quantify how specific factors (eg, risk and normative information) modulate this spread rather than to isolate the precise underlying psychological mechanisms of behavioural contagion (eg, distinguishing between simple imitation vs normative pressure).

### Implications for practice

Findings from the Epi-Q study have several major practical implications. First, they can inform public health communication strategies by clarifying whether individuals are more responsive to information about local, socially proximal risks versus population-level, aggregate statistics. If local information is more effective in motivating compliance, as suggested by construal level theory^13^ or egocentric bias in risk perception^40^, then messaging could be tailored accordingly. Second, this study will shed light on the relative importance of perceived risk versus social norms, which could guide the design of interventions that leverage peer influence to promote protective behaviours. Critically, by testing interventions in a networked context, our findings can help design strategies that mitigate the negative effects of stigma and social isolation, which during COVID-19 fostered mistrust and non-compliance^6 7 38^. Third, the methodological framework itself (epigames implemented via mobile apps) offers a scalable, customisable and low-cost tool for studying human behavioural responses during health emergencies. Beyond quarantine, the same approach can be adapted to examine other key interventions such as vaccination, mask use and business closures in a way that accounts for the social and cultural dimensions of these actions.

### Ethics and dissemination

#### Ethical approval and consent

This study protocol has been reviewed and approved by the Institutional Review Board (IRB) at UMass Chan Medical School (eIRB Study ID: STUDY00002465). Prior to participating, all participants will complete an electronic informed consent process within the custom smartphone app. The onboarding flow details the study design, experimental procedures, potential risks, data protection protocols and intended use of data for human subjects research. Participants must acknowledge reading and understanding these provisions via an explicit opt-in checkbox and timestamp in the app before any data collection commences. Participation is entirely voluntary, and participants can withdraw from the study at any time via a dedicated option within the app settings, which triggers the immediate and permanent deletion of their stored data.

#### Ethical approval and consent

To ensure complete anonymity, no personally identifiable information (PII)—such as names, email addresses, phone numbers, location tracking or IP addresses—will be collected or stored. on consent, each device is assigned a randomly generated 128-bit unique ID. All app logs, survey responses and Bluetooth proximity records are linked exclusively to this non-identifiable ID.

Although Android OS permissions require location services to execute Bluetooth Low Energy scanning, raw GPS and location data are neither captured nor stored by the application. All data transmissions between mobile devices and the backend (deployed on either secure Amazon Web Services cloud infrastructure or a dedicated self-hosted server) are encrypted both in transit and at rest.

### Compensation plans

To account for varying experimental contexts and study scales while preventing behavioural bias, compensation structures are tailored across study deployments:

- Direct fixed compensation: in smaller or longer-duration study cohorts, participants will receive digital gift cards (typically valued between US$5 and US$20) delivered directly and anonymously through the app on game completion.
- Lottery system: for larger-scale deployments (eg, >500 participants), entry into a point-proportional lottery draw for digital gift cards will be used to maintain cost-effectiveness while preserving behavioural incentive structures.
- Uncompensated studies: in specific trials where financial incentives might confound intrinsic social motivators (eg, studies measuring pure altruism or prosocial behaviours) or in professional settings (eg, academic conferences), participation will be uncompensated.

All compensation terms and distribution mechanisms are fully disclosed to participants during the electronic consent process prior to enrolment.

### Dissemination strategy

Study findings will be published in open-access, peer-reviewed scientific journals covering infectious disease epidemiology, behavioural science, social network analysis and digital health. Results will also be presented at national and international academic conferences.

While individual-level results will not be returned to participants because of data anonymisation, aggregate findings and key scientific takeaways will be summarised in public-facing formats. We plan to collaborate with science communicators to produce accessible visual summaries and blog posts for participants and general audiences. Fully de-identified behavioural and network datasets, along with custom analytical code (R and Python), will be deposited in open-access repositories (such as the Open Science Framework) on publication of primary outcomes.

## Supplementary material

10.1136/bmjopen-2026-116726 online supplemental file 1

## Data Availability

Data are available upon reasonable request.
