# Supplementary material for: Effect of local versus global risk and normative information on voluntary quarantine adoption: protocol for a 2×2 randomised controlled trial leveraging app-based epigames across diverse congregate settings (The Epi-Q Study)
Source: BMJ Open. 2026 Oct 8;16(10):e116726. doi: 10.1136/bmjopen-2026-116726 (PMC13656103; doi:10.1136/bmjopen-2026-116726)
Supplement: Supplementary file 1 [file bmjopen-2026-116726.reviewer_comments.pdf]

PEER REVIEW HISTORY

BMJ Open publishes all reviews undertaken for accepted manuscripts. Reviewers are asked to complete a checklist review form and are provided with free text boxes to elaborate on their assessment. These free text comments are reproduced below.

ARTICLE DETAILS

Title (Provisional)

Effect of local versus global risk and normative information on voluntary quarantine adoption: protocol for a 2x2 randomized controlled trial leveraging app-based epigames across diverse congregate settings (The Epi-Q Study)

Authors

McCool, Estelle; Mkandawire, Winnie W; Musa, Salihu S; Chen, Shi; Moley, Laura; Robbins Schug, Gwen; Bowman, Nick; Nowak, Sarah A; Drake, John; Drury, J; Fowler, Adam; Mills, Melinda; Fraser, Christophe; Ferretti, Luca; Swallow, Ben; Panovska Griffiths, Jasmina; Colubri, Andres

---

VERSION 1 - REVIEW

**Reviewer**	**1**
**Name**	**Gray, Lesley**
**Affiliation**	**University of Otago, Primary Health Care and General Practice**
**Date**	**18-Jul-2026**
**COI**	**None**

This is an interesting (and indeed novel) Protocol relative to quarantine behaviours documenting a planned study. I do hope the the Bluetooth proximity function delivers reliable real-time proximity data of the participants to simulate some form of real time quarantine and I look forward to reading the results. There are a couple of items that need to be completed:

- I could not see the planned dates of the study - these should be included in the manuscript.

- While trial registration is included I could not see a trial checklist with the manuscript.

The authors may be interested in a couple of additional papers (no obligation to consider including, merely provided for information):

- Turnbull et al. https://doi.org/10.1016/j.lanwpc.2021.100351

- Gray et al. https://doi.org/10.1080/03036758.2021.1879179

VERSION 1 - AUTHOR RESPONSE

Reviewer: 1
Ms. Lesley Gray, University of Otago
Comments to the Author:
This is an interesting (and indeed novel) Protocol relative to quarantine behaviours documenting a planned study. I do hope the the Bluetooth proximity function delivers reliable real-time proximity data of the participants to simulate some form of real time quarantine and I look forward to reading the results.

We are grateful to the reviewer for the positive comments on the protocol and the planned study.

There are a couple of items that need to be completed:
- I could not see the planned dates of the study - these should be included in the manuscript.

We are planning to run the study across 2-3 settings and cohorts. We are anticipating running these in the period March 2027-December 2027, but the exact dates are not specified yet. In response to the reviewer's comment, we have added the timeline March 2027-December 2027 as the dates of the study.

- While trial registration is included I could not see a trial checklist with the manuscript.

We appreciate this comment. We have downloaded a SPIRIT checklist from https://www.consort-spirit.org/, completed it and have now included it as a separate document with the resubmission.

The authors may be interested in a couple of additional papers (no obligation to consider including, merely provided for information):
- Turnbull et al. https://doi.org/10.1016/j.lanwpc.2021.100351
- Gray et al. https://doi.org/10.1080/03036758.2021.1879179

Many thanks for highlighting these papers. We have found them useful and included them in the motivation for our work, referring to their main aims.

Reviewer: 1
If you have selected 'Yes' above, please provide details of any competing interests.: Not applicable
