# Supplementary material for: Effect of local versus global risk and normative information on voluntary quarantine adoption: protocol for a 2×2 randomised controlled trial leveraging app-based epigames across diverse congregate settings (The Epi-Q Study)
Source: BMJ Open. 2026 Oct 8;16(10):e116726. doi: 10.1136/bmjopen-2026-116726 (PMC13656103; doi:10.1136/bmjopen-2026-116726)
Supplement: Supplementary file 2 [file bmjopen-16-10-s001.docx]

**Supplementary Materials**

**S1.** Survey of baseline real-life perceptions to be administered immediately after recruitment, once they the install the app, open it for the first time and consent to participate in the study:

1. “How likely do you think it is that you will get infected with a circulating respiratory virus (e.g., influenza, cold, or COVID) in the next three months? Please use a scale from 1 to 6, where 1 means virtually impossible and 6 means almost certain.”
2. “If you were to get infected with a circulating respiratory virus (e.g., influenza, cold, or COVID) in the next three months, how serious do you think the impact would be on your health? Please use a scale from 1 to 6, where 1 means no impact at all and 6 means a very significant impact on your health.”
3. “How confident are you that you would be able to successfully follow a home quarantine if you were asked to quarantine in the near future due to exposure to a new virus with potentially serious health effects (such as covid at the beginning of the pandemic)? Please use a scale from 1 to 6, where 1 means you are not confident at all and 6 means you are completely confident that you would be able to follow the quarantine.”
4. “How effective do you think following a home quarantine would be in preventing the spread of a new virus with potentially serious health effects (such as covid at the beginning of the pandemic)? Please use a scale from 1 to 6, where 1 means you think it is not effective at all and 7 means you think it is extremely effective.”
5. “How likely you would be to follow a home quarantine if you knew that most people around you (e.g., friends, family, co-workers) would also quarantine if exposed to or infected with a new virus with potentially serious health effects (such as covid at the beginning of the pandemic)? Please use a scale from 1 to 6, where 1 means you would not quarantine at all and 6 means you would be extremely likely to quarantine.”
6. “How much do you identify with the group of people you’ll be playing the game with (e.g., other participants on your campus or at this event)? Please use a scale from 1 to 6, where 1 means not at all and 6 means you identify with them very much.”

**S2.** Survey of anticipated game-related perceptions to be administered during the calibration stage:

1. “How likely do you think it is that you will get infected with the virtual pathogen while playing with the app during the next two weeks? Please use a scale from 1 to 6, where 1 means virtually impossible and 6 means almost certain.”
2. “If you were to get infected with the virtual pathogen while playing with the app during the next two weeks, how much would you be concerned about it given the possibility of winning a reward with the point-based lottery at the end of the game? Please use a scale from 1 to 6, where 1 means you will not be concerned at all and 6 means that you will be extremely concerned.”
3. “How confident you are that you will be able to successfully follow the in-game quarantine guidelines if you are asked to quarantine while playing with the app, using a scale from 1 to 6, where 1 means you are not confident at all and 6 means you are completely confident that you will be able to follow the quarantine.”
4. “Please tell us how effective you think in-game quarantine is in preventing the spread of the virtual pathogen while playing with the app, using a scale from 1 to 6, where 1 means you think it is not effective at all and 6 means you think it is extremely effective.”
5. “Please tell us how likely you are to quarantine while playing with the app if you know that most other players are also choosing to quarantine when exposed or infected, using a scale from 1 to 6, where 1 means you are not likely to quarantine at all and 6 means you are extremely likely to quarantine.”

**S3.** Exit survey of post-game perceptions to be administered immediately after the game has ended:

1. “Now that the game is over, how likely do you think it was that you would get infected with the virtual pathogen during the game? Please use a scale from 1 to 6, where 1 means virtually impossible and 6 means almost certain.”
2. “If you had been infected during the game, how serious do you think the consequences would have been in terms of your chance of winning the final reward? Please use a scale from 1 to 6, where 1 means no impact at all and 6 means very significant impact.”
3. “How closely did your quarantine decisions during the game reflect what you would actually do in a real-life outbreak with similar risks and rewards? Please use a scale from 1 to 6, where 1 means not at all similar and 6 means exactly what I’d do in real life.”
4. “How easy or difficult was it to process the information you received during the game? Please use a scale from 1 to 6, where 1 means very easy and 6 means very difficult/overwhelming.”
5. “To what extent did seeing others choose to quarantine make you more likely to do the same? Please use a scale from 1 to 6, where 1 means not at all and 6 means very strongly influenced me.”
6. “To what extent did seeing that your close contacts were infected or not quarantining make you more concerned about getting infected yourself?” Please use a scale from 1 to 6, where 1 means not at all concerned and 6 means very concerned.”
